# Epidemiology and Clinical Features of *Candida* Bloodstream Infections: A 10-Year Retrospective Study in a Korean Teaching Hospital †

**DOI:** 10.3390/jof11030217

**Published:** 2025-03-12

**Authors:** Shi Nae Yu, Sun In Hong, Jung Wan Park, Min Hyok Jeon, Oh Hyun Cho

**Affiliations:** Division of Infectious Diseases, Department of Internal Medicine, Soonchunhyang University Cheonan Hospital, Soonchunhyang University College of Medicine, 31, Suncheonhyang 6-gil, Dongnam-gu, Cheonan 31151, Republic of Korea; zolzoly@schmc.ac.kr (S.N.Y.); 101257@schmc.ac.kr (S.I.H.); splendidmagic@schmc.ac.kr (J.W.P.); yacsog@schmc.ac.kr (M.H.J.)

**Keywords:** *Candida*, candidemia, epidemiology

## Abstract

*Candida* species are major pathogens of bloodstream infections (BSIs) in hospitalized patients, with high mortality. This study examined *Candida* species distribution, clinical characteristics, and the mortality of patients with *Candida* BSIs. Adult patients (≥16 years) with *Candida* BSIs at a teaching hospital (2014–2023) were retrospectively reviewed. Over 10 years, 487 *Candida* isolates were obtained from 462 patients. *C. albicans* was the most frequent (38.2%), followed by *C. glabrata* (21.1%), *C. parapsilosis* (20.5%), and *C. tropicalis* (13.3%). The annual incidence of *Candida* BSIs remained stable (*p* = 0.525). However, non-*albicans* species BSIs increased 1.61-fold compared to *C. albicans* (95% CI: 1.19–2.19, *p* = 0.002). Fluconazole-non-susceptible *Candida* isolates increased after 2021 (*p* = 0.040). The overall 30-day mortality was 40.6%. In the multivariate analysis, a high Charlson comorbidity index (aHR: 1.20, 95% CI: 1.07–1.35, *p* = 0.001) and high SOFA score (aHR: 1.12, 95% CI: 1.02–1.23, *p* = 0.022) were the strongest predictors of 30-day mortality. Meanwhile, *C. parapsilosis* BSIs (aHR: 0.46, 95% CI: 0.22–0.99, *p* = 0.047) and central venous catheter removal at any time (aHR: 0.22, 95% CI: 0.13–0.37, *p* < 0.001) were associated with reduced 30-day mortality. The mortality of patients with *Candida* BSIs was mainly determined by disease severity, while catheter removal was associated with improved survival.

## 1. Introduction

*Candida*, one of the most important causative agents of bloodstream infections (BSIs) in hospitals, is associated with high morbidity and mortality [1,2]. *Candida* species is the fourth most common cause of nosocomial BSIs in the U.S. [3]. It has been the most frequent cause of BSIs in intensive care units (ICUs) in Korea since 2013 [4]. *Candida* BSI is common in patients admitted to ICUs or those who are immunocompromised. Identified risk factors include the presence of central venous catheters, surgical procedures (especially abdominal surgery), total parenteral nutrition, broad-spectrum antibiotic therapy, and disruptions to the integrity of the skin or mucosal barriers [5,6].

Although *Candida albicans* has been reported as the most common cause of *Candida* BSIs, recent studies have shown an increasing proportion of non-*albicans Candida* BSIs [6,7,8]. While there might be regional epidemiologic differences, some studies have reported an increase in fluconazole-resistant isolates among non-*albicans Candida* species from BSIs, particularly *C. auris*, *C. glabrata* (*Nakaseomyces glabratus*), and *C. parapsilosis* [9,10,11]. In Korea, the proportion of non-*albicans Candida* BSIs has exceeded that of *C. albicans* since 2006 [12]. The selection of appropriate antifungal agents for *Candida* BSIs necessitates epidemiologic data on the distribution of *Candida* species and their antifungal resistance profiles. Therefore, ongoing surveillance data on the distribution of *Candida* species from BSIs and antifungal susceptibility are important not only at the national level, but also at the institutional level. This study aimed to analyze clinical features, epidemiological characteristics, and antifungal susceptibility patterns of *Candida* BSIs at our hospital from 2014 to 2023. Risk factors for 30-day mortality were also examined.

## 2. Methods

This retrospective cohort study was conducted at an 890-bed teaching hospital, including 51 beds in three intensive care units (ICUs), in the Republic of Korea. Electronic medical records of all adult patients aged ≥ 16 who were diagnosed with *Candida* BSIs between January 2014 and December 2023 were reviewed. This study was approved by the Institutional Review Board (IRB) of Soonchunhyang University Cheonan Hospital (IRB approval No. 2024-05-030).

### 2.1. Definitions and Data Collection

All *Candida* BSIs were retrieved from the microbiology laboratory database. *Candida* BSI was defined as the isolation of *Candida* species from at least one blood culture in patients with compatible clinical symptoms or signs of infection. Among patients with multiple *Candida* BSI episodes during the same hospitalization period, only the first episode for each patient was included. Patients with multiple *Candida* species identified from the same blood cultures, patients with incomplete medical records, and patients whose outcomes were unknown due to transfer to other hospitals were excluded from clinical outcome analysis.

The following data were collected from the electronic medical records of each patient: demographics, underlying diseases, risk factors for *Candida* BSIs, severity of illness, laboratory results on the day of *Candida* BSI onset, focus of infection, complications, the time from drawing the first blood culture positive for *Candida* species to removal of central venous catheter (CVC), antifungal treatment, 7-day mortality, and 30-day mortality. The severity of illness was evaluated with the sequential organ failure assessment (SOFA) score, the Charlson comorbidity index, and the presence of septic shock.

CVC-related *Candida* BSI was defined as isolation of the same *Candida* species from blood and catheter tip cultures, or when the same species was isolated from both CVC and peripheral vein blood cultures, with positive values for differential time to positivity (TTP) [13]. The gastrointestinal tract was considered as the source of *Candida* BSI if patients had signs or symptoms related to the gastrointestinal tract prior to the onset of candidemia which did not have any other sources. The urinary tract was considered to be the portal of entry for patients with obstructive uropathy and evidence of urinary tract infection caused by the same species of *Candida*. Cases that did not meet the above-mentioned criteria were classified as primary cases. An appropriate empirical antifungal treatment was considered when at least one antifungal agent with in vitro activity was started within 48 h after the first blood culture was performed. Persistent *Candida* BSI was defined as isolation of the same *Candida* species ≥ 5 days after initiation of antifungal therapy. Early CVC removal was considered when the CVC was removed within 48 h from drawing the first blood culture positive for *Candida* species. The incidence of *Candida* BSIs was determined by the ratio of the total number of *Candida* BSI episodes per 1000 patient-days.

### 2.2. Microbiological Methods

Blood culture bottles were incubated in a BacT/ALERT 3D system (BioMérieux Inc., Marcy l’Étoile, France) for five days. *Candida* species were identified using a VITEK-2 automated system (BioMérieux Inc.) until March 2019. Thereafter, identification was performed using matrix-assisted laser desorption/ionization–time-of-flight mass spectrometry (MALDI-TOF MS; Microflex LT, Bruker Daltonics, Bremen, Germany). Antimicrobial susceptibility testing was performed using a VITEK-2 automated system (BioMérieux Inc.) until March 2019 and the Sensititre YeastOne system (Thermo Fisher Scientific Inc., Cleveland, OH, USA) thereafter, following the manufacturer’s recommendations. Interpretation of susceptibility was performed according to clinical breakpoint defined by the Clinical and Laboratory Standards Institute (CLSI) [14].

### 2.3. Statistical Analysis

Continuous variables were compared using the Mann–Whitney *U*-test. Categorical variables were compared using the chi-square test or Fisher’s exact test. Comparisons of continuous variables between different *Candida* species were performed using the Kruskal–Wallis test. The change in the annual incidence of *Candida* BSIs during the study period and difference in incidence between *C. albicans* and non-*albicans* BSIs were analyzed using negative binominal regression analysis. The Kaplan–Meier method was used to calculate the 30-day survival probability of each *Candida* species. Differences between survival curves were compared with the log-rank test. Cox regression analysis was used to evaluate risk factors for 30-day mortality. Variables with *p* < 0.1 after univariate analysis and factors relevant to outcomes were entered into a multivariable model. All *p*-values were two-tailed and *p* < 0.05 was considered statistically significant. All statistical analyses were performed using R (version 4.4.1) and R Studio (version 2024.04.2+764) software.

## 3. Results

### 3.1. Incidence of Candida BSIs and Antifungal Susceptibility Profiles

**Figure 1:**
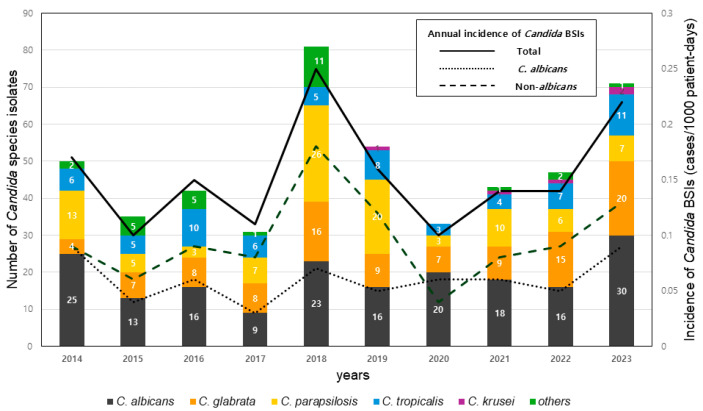
Trends in annual incidence of *Candida* bloodstream infections and distribution of *Candida* isolates identified over the 10-year study period.

During the 10-year study period, 487 *Candida* isolates were obtained from 462 patients with *Candida* BSIs. *C. albicans* was the most frequent species (n = 186, 38.2%), followed by *C. glabrata* (n = 103, 21.1%), *C. parapsilosis* (n = 100, 20.5%), *C. tropicalis* (n = 65, 13.3%), *C. guilliermondii* (*Meyerozyma guilliermondii*) (n = 7, 1.4%), *C. famata* (*Debaryomyces hansenii*) (n = 6, 1.2%), and *C. krusei* (*Pichia Kudriavzevii*) (n = 5, 1.0%). Other *Candida* species accounted for 3.1% (n = 15) including *C. pelliculosa* (*Wickerhamomyces anomalus*) (n = 5), *C. dubliniensis* (n = 3), *C. lusitaniae* (*Clavispora lusitaniae*) (n = 2), *C. intermedia* (n = 2), *C. fabianii* (*Cyberlindnera fabianii*) (n = 1), *C. utilis* (*Cyberlindnera jadinii*) (n = 1), and *C. sake* (n = 1) (Figure 1). Overall, the annual incidence of *Candida* BSIs was 0.15 episodes per 1000 patient-days. Annual incidences of *Candida* BSIs were not significantly different during the study period (*p* = 0.525). The trend in annual *Candida* BSI incidence showed a 1.61-fold (95% CI: 1.19–2.19, *p* = 0.002) increase in non-*albicans* species compared to *C. albicans* over the study period.

Table 1 shows antifungal susceptibility results for 456 *Candida* isolates from BSIs. These isolates had susceptibility tests available. When resistant (R) and dose-dependent susceptible/intermediate (SDD/I) categories were considered as non-susceptible, the overall fluconazole non-susceptibility rate was 26.5% (121/456). Fluconazole-non-susceptible *Candida* isolates showed an increasing trend from 2021 (*p* = 0.040), attributed to increases in *C. glabrata* and *C. krusei* BSIs (Figure 1 and Supplementary Figure S1). Excluding *C. glabrata* isolates due to the lack of established clinical breakpoints for voriconazole in the CLSI, the voriconazole non-susceptibility rate was 5.4% (19/355). Resistance to micafungin and amphotericin B was rare, with 1.1% (5/456) and 0.9% (4/456) of isolates showing resistance to micafungin and amphotericin B, respectively. The rate of fluconazole resistance was 1.6% (3/186) in *C. albicans* and 1% (1/95) in *C. parapsilosis*. Meanwhile, the rate of resistance of *C. tropicalis* to fluconazole and voriconazole was 4.7% (3/64) each. SDD/I rates for fluconazole and voriconazole were 6.3% (4/64) and 15.6% (10/64), respectively. Three *Candida* isolates exhibited multidrug resistance: one *C. parapsilosis* to both fluconazole and amphotericin B, one *C. glabrata* to both fluconazole and micafungin, and one *C. krusei* to both azoles and amphotericin B.

### 3.2. Clinical Features of Patients with Candida BSIs

A total of 409 patients were included for the analysis of clinical features of *Candida* BSIs after excluding cases with multiple *Candida* species isolated from the same patients (n = 15), those with clinically insignificant isolation (n = 17), those who were transferred to other hospitals (n = 6), and those with incomplete medical records (n = 15). Demographic data, clinical characteristics, and outcomes according to *Candida* species are shown in Table 2. The median age of all patients was 71 years (IQR: 61–79 years). There were 222 (54.3%) males. Most of them had underlying diseases, including solid tumor (n = 228, 55.7%) and diabetes mellitus (n = 155, 37.9%). There were 325 cases (79.5%) of nosocomial *Candida* BSIs, with 91 cases (22.2%) occurring in the ICUs. At the time of *Candida* BSIs, 321 (78.5%) patients had had a CVC and 175 (42.8%) received total parenteral nutrition. The most common source of BSI was primary BSI (n = 242, 59.2%), followed by catheter-related bloodstream infection (CRBSI) (n = 119, 29.1%), urinary tract infections (n = 24, 5.9%), and intra-abdominal infections (n = 22, 5.4%). Of 154 patients who underwent fundus examination, 8 (5.1%) had ocular candidiasis. Antifungal treatment was administered to 351 (85.8%) patients. Of them, 203 (57.8%) received adequate empirical therapy.

Since *C. albicans*, *C. parapsilosis*, *C. glabrata*, and *C. tropicalis* BSIs were predominant (94.6%) in this study, we compared the clinical characteristics and outcomes of those with *C. albicans* and those with the other three *Candida* species (Supplementary Table S1), as these comparisons are clinically more relevant. Patients with *C. parapsilosis* BSIs had lower 7-day and 30-day mortality (*p* = 0.005 and *p* = 0.004, each), a shorter ICU stay at *Candida* BSI onset (*p* = 0.021), less surgery (*p* = 0.021), less colonization before BSI (*p* = 0.004), and lower severity, including septic shock (*p* = 0.004) and SOFA score (*p* = 0.012). Meanwhile, they received more chemotherapy (*p* = 0.012), had more CRBSI (*p* < 0.001), and had more persistent *Candida* BSIs (*p* = 0.004). Patients with *C. glabrata* BSIs had a longer TTP (*p* = 0.001) and more intra-abdominal infection (*p* = 0.001), but less CRBSI (*p* = 0.009). Although they often did not receive appropriate empirical antifungal therapy (*p* = 0.010), with delayed removal of CVC (*p* = 0.002), they showed no significant difference in mortality rate compared to patients with *C. albicans* BSIs. Patients with *C. tropicalis* BSIs showed clinical characteristics and outcomes similar to those with *C. albicans*, except that they had a shorter TTP (*p* = 0.001). The results of pairwise multiple comparisons are also presented in Supplementary Table S2, where statistical significance was determined using *p* < 0.005 based on the Bonferroni adjustment.

### 3.3. Analysis of 30-Day Mortality Predictors

**Figure 2:**
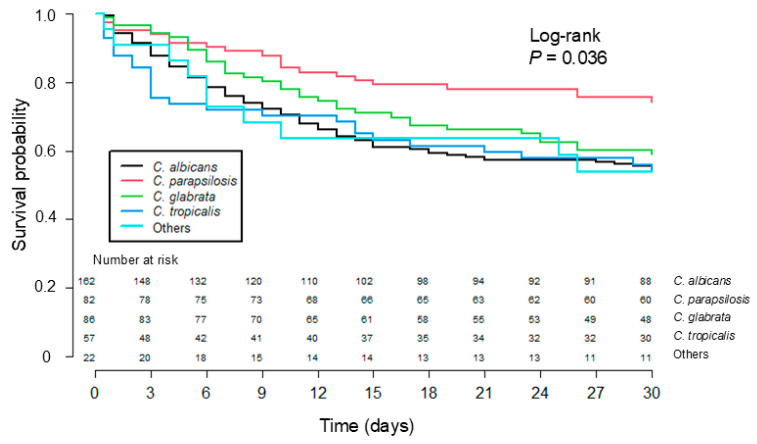
Kaplan–Meier survival curve showing 30-day mortality of patients with *Candida* bloodstream infections caused by different *Candida* species.

The overall mortality was 21.5% at 7 days and 40.6% at 30 days. Patients with *Candida* BSIs caused by different *Candida* species showed significantly different 30-day survival probabilities (Figure 2, *p* = 0.036), which were related to a low 30-day mortality of *C. parapsilosis* BSIs (*p* = 0.003). Results of univariate and multivariate analyses for predictors of 30-day mortality of patients with *Candida* BSIs are shown in Table 3. A high Charlson comorbidity index (adjusted hazard ratio [aHR]: 1.20, 95% CI: 1.07–1.35, *p* = 0.001) and high SOFA score (aHR: 1.12, 95% CI: 1.02–1.23, *p* = 0.022) were independently associated with 30-day mortality. Meanwhile, *C. parapsilosis* BSIs (aHR: 0.46, 95% CI: 0.22–0.99, *p* = 0.047) and CVC removal at any time (aHR: 0.22, 95% CI: 0.13–0.37, *p* < 0.001) were significantly associated with reduced 30-day mortality.

## 4. Discussion

In this study, we investigated the distribution of *Candida* species isolated from bloodstream infections and their antifungal susceptibility patterns over a 10-year period in a teaching hospital. *C. albicans* was the most common species, accounting for 38.2% of cases. The incidence of *Candida* BSIs remained stable over the study period. However, the proportion of non-*albicans* BSIs showed a slightly increasing trend. The proportion of *C. glabrata* also showed an increase among non-*albicans* species from 2021. Although *C. albicans* is the most prevalent species in most regions of the world, an increasing proportion of non-*albicans Candida* BSIs has been observed [6]. While differences exist among studies, *C. glabrata* has been reported as the most common non-*albicans* species in both the US and the Asia–Pacific region [15,16]. In most parts of Europe, *C. glabrata* is also prevalent, but in Italy and Turkey, *C. parapsilosis* has been reported as the dominant non-*albicans* species [11]. In contrast, in South America, *C. parapsilosis* and *C. tropicalis* are more frequently observed [15]. According to a report from the Korean National Healthcare-Associated Infection Surveillance System (KONIS), there were 2248 cases of *Candida* BSIs in ICUs from 2006 to 2017, with an incidence rate of 0.25 cases per 1000 PD [4]. *C. albicans* was the most prevalent species, accounting for 39.9% of cases, followed by *C. tropicalis* (20.2%) and *C. parapsilosis* (18.2%), while *C. glabrata* showed an increasing trend from 8.9% in 2006 to 17.9% in 2017 [4]. Another study using data from nine sentinel hospitals (Kor-GLASS program) in Korea during 2020–2021 reported that the average incidence of *Candida* BSIs was 0.16 cases per 1000 PD (range, 0.11–0.22 cases per 1000 PD) [17]. Among 766 isolates from *Candida* BSIs, *C. albicans* was the most common (45.4%), followed by *C. tropicalis* (17.6%), *C. glabrata* (17.4%), and *C. parapsilosis* (14.1%) [17]. In our study, the potential outbreak of *C. parapsilosis* BSIs in 2018 and 2019 cannot be ruled out. Despite this possible outbreak, our data showed a similar incidence rate of *Candida* BSIs and a comparable proportion of non-*albicans* BSIs to those observed in two surveillance datasets from Korea [4,17].

According to the SENTRY Antifungal Surveillance Program, fluconazole resistance among non-*albicans Candida* species varied by region. Fluconazole resistance in *C. glabrata* was high in North America, reaching 10.6%, while in the Asia–Pacific region, *C. tropicalis* showed a high fluconazole resistance rate of 9.2% [15]. In the United States, fluconazole resistance in *C. glabrata* demonstrated a steady increase over 20 years [15,16]. The rate of micafungin resistance in the Asia–Pacific region was lower than that in North America, with *C. glabrata* showing a resistance rate of 0.4% while other *Candida* species showed no resistance [16]. Meanwhile, the Kor-GLASS program assessing isolates collected in 2020 and 2021 found that, among 741 BSI isolates, *C. albicans* exhibited no resistance to fluconazole, while *C. parapsilosis*, *C glabrata*, and *C. tropicalis* showed resistance rates of 5.6%, 5.3%, and 2.2%, respectively. Only one *C. glabrata* isolate demonstrated resistance to caspofungin [17]. Another study conducted in 19 tertiary hospitals in South Korea reported that the proportion of *C. glabrata* in 1158 *Candida* BSI cases steadily increased from 11.7% in 2008 to 23.9% in 2018. However, fluconazole resistance remained relatively stable throughout the study period, with an average rate of 5.7% [18]. In our study, an increasing trend in fluconazole-non-susceptible *Candida* isolates was observed after 2021, attributed to increases in *C. glabrata* and *C. krusei*. However, similar to other studies in South Korea, azole resistance was observed only sporadically throughout the study period. The relatively high azole resistance rate of *C. tropicalis* was concerning, similar to that in the Asia–Pacific region in the SENTRY data, while resistance rates to micafungin and amphotericin B were approximately 1%, slightly higher than those in the reports of the Kor-GLASS program [4,17]. However, as susceptibility results in this study were based on data obtained using commercial antifungal susceptibility tests, some discrepancies might exist compared to data obtained through standardized microdilution methods [19].

Previous studies investigating the clinical characteristics of BSIs caused by each *Candida* species or comparing BSIs caused by *C. albicans* with those caused by non-*albicans* species have suggested that BSIs caused by each non-*albicans* display distinct clinical features [12,20,21]. In the present study, *C. glabrata* showed a longer TTP than *C. albicans*. A longer TTP was frequently associated with intra-abdominal infection sources. Notably, the prolonged TTP for *C. glabrata* found in the present study is consistent with previous studies [22,23]. Although patients with *C. tropicalis* BSIs exhibited a shorter TTP than those with *C. albicans* infections, mortality rates were similar in our data. Some studies have reported that the mortality rate is the highest in patients with *C. tropicalis* BSIs compared to those with BSIs and other *Candida* species [12,24,25]. Considering that *C. tropicalis* BSIs are more prevalent in patients with neutropenia or hematologic malignancies and that a shorter TTP allows for earlier administration of empirical antifungal agents or prompt removal of central venous catheters, further investigation of mortality data is warranted [21]. In prior research, *C. parapsilosis* BSI has been associated with lower mortality and complication rates compared to *C. albicans* BSI, along with a higher incidence of CRBSI [25,26,27]. Our study similarly found that patients with *C. parapsilosis* BSIs had lower mortality rates without endophthalmitis, in contrast to those with *C. albicans* infections. However, CRBSIs and persistent fungemia were more frequently observed in patients with *C. parapsilosis* BSIs. In the multivariate analysis, the 30-day mortality was lower for patients with *C. parapsilosis* BSIs in the present study. However, given that this patient group had more than twice the rate of CRBSIs and lower severity compared to other groups, confounding variables might not have been sufficiently adjusted. Given that CRBSIs are generally considered low-mortality BSIs [28], further well-designed comparative studies among *Candida* BSIs are needed.

In this study, independent risk factors associated with 30-day mortality in patients with *Candida* BSIs were identified as a high Charlson comorbidity index (CCI) and high SOFA score, while removal of CVC at any time was associated with a reduction in mortality. In the univariate analysis, early removal of CVCs and early initiation of adequate empirical antifungal therapy were linked to reduced mortality. However, these factors did not show statistically significant differences in the multivariate analysis. Although the Infectious Diseases Society of America (IDSA) guidelines recommend early empirical antifungal therapy and CVC removal as soon as possible for *Candida* BSIs, evidence on their impact in reducing candidemia mortality remains mixed, varying by patient severity, underlying health conditions, and study design [19,29,30]. Definitions of early empirical antifungal administration or catheter removal timeframes range from within 24 h to within 3 days. Starting points can also vary from the time of blood culture sampling to the time of positive culture identification, complicating the direct comparison of outcomes [20,31,32,33]. Lee et al. have reported that early removal of CVC is associated with reduced mortality only in patients with a CCI score of less than 4, suggesting that baseline condition at the onset of candidemia is crucial [34]. In this study, 86% of included patients had a CCI score of 4 or higher. Thus, well-designed follow-up studies are needed to draw definitive conclusions regarding the issue of the impact of early empirical antifungal therapy and early removal of CVCs on mortality reduction. Meanwhile, this study, along with other studies, found that CVC removal (irrespective of timing) was associated with reduced mortality [25,35]. However, in patients with limited life expectancy due to underlying conditions, CVCs are often not removed. Since such patients might have been overrepresented in studies, this could potentially lead to an overestimation of the impact on results [36].

This study has several limitations. First, this is a retrospective cohort study, which might be subject to selection and observation biases that could affect study results. Although we attempted to adjust for confounding factors using multivariate analysis, unmeasured or residual confounders may still exist and influence our findings. The survival probability of patients with *Candida* BSIs may be overestimated because competing risks, such as nonfungal sepsis, were not taken into account. Treatment for *Candida* BSI was determined by the attending physician, leading to non-standardized treatment. In some cases, serial follow-up blood cultures were not performed or TTP was not recorded, which prevented the inclusion of these key variables in the multivariate analysis. Until March 2019, *Candida* species were identified using the VITEK-2 system, which may have led to misidentification. In particular, *C. auris*, an emerging and significant healthcare-associated fungal pathogen, was not detected in our dataset, but the possibility of misidentification cannot be ruled out [37]. Lastly, as a single-center study, the epidemiology and resistance patterns of *Candida* species observed in this study might not be representative of the nationwide situation.

In conclusion, our study showed that the annual incidence of *Candida* BSIs was similar. However, non-*albicans* species seemed to increase over the study period, with fluconazole-non-susceptible isolates increasing from 2021. Given the role of antifungal exposure in the emergence of non-*albicans* or resistant strains, judicious antifungal use is imperative [38]. The mortality of patients with *Candida* BSIs was high, at 40.6%, while the mortality rate for those with *C. parapsilosis* BSI was lower than that for those with other *Candida* species. Underlying conditions such as CCI and the SOFA score were significant predictors of mortality. Removal of catheters at any time had a positive impact on reducing mortality.

## Figures and Tables

**Table 1:** Antifungal susceptibility results of *Candida* species isolates from blood cultures.

	*Candida albicans* (n = 186)	*Candida parapsilosis* (n = 95)	*Candida glabrata* ^a^ (n = 101)	*Candida tropicalis* (n = 64)	*Candida krusei* (n = 5)	*Candida guilliermondii* (n = 5)	Total (n = 456)
Fluconazole							
S	179 (96.2)	94 (99.0)	0	57 (89.1)	0	5 (100)	335 (73.5)
SDD/I	4 (2.1)	0	95 (94.1)	4 (6.3)	0	0	103 (22.6)
R	3 (1.6)	1 (1.0)	6 (5.9)	3 (4.7)	5 (100)	0	18 (3.9)
Voriconazole							
S	182 (97.8)	95 (100)	–	51 (79.7)	3 (60.0)	5 (100)	336 (94.6) ^b^
SDD/I	0	0	–	10 (15.6)	0	0	10 (2.8) ^b^
R	4 (2.1)	0	–	3 (4.7)	2 (40.0)	0	9 (2.5) ^b^
Micafungin							
S	186 (100)	93 (97.9)	98 (97.0)	64 (100)	5 (100)	5 (100)	451 (98.9)
SDD/I	0	0	0	0	0	0	0
R	0	2 (2.1)	3 (3.0)	0	0	0	5 (1.1)
Amphotericin B							
S	185 (99.5)	93 (97.9)	101 (100)	64 (100)	4 (80.0)	5 (100)	452 (99.1)
SDD/I	0	0	0	0	0	0	0
R	1 (0.5)	2 (2.1)	0	0	1 (20.0)	0	4 (0.9)

Data are presented as number (%). Abbreviations: S, susceptible; SDD/I, susceptible dose-dependent/intermediate; R, resistant. ^a^ Clinical breakpoint for voriconazole to *C. glabrata* is not established in the Clinical and Laboratory Standards Institute. ^b^ The 101 *C. glabrata* isolates were excluded when calculating the proportion of voriconazole-susceptible or -non-susceptible isolates.

**Table 2:** Baseline and clinical characteristics of patients with *Candida* bloodstream infections.

	Total	*C. albicans*	*C. parapsilosis*	*C. glabrata*	*C. tropicalis*	Others ^a^	
Variable	(n = 409)	(n = 162)	(n = 82)	(n = 86)	(n = 57)	(n = 22)	*p* ^b^
Age (years)	71 (61–79)	70 (62–78)	71 (61–79)	74 (63–80)	68 (58–75)	77 (62–79)	0.281
Male	222 (54.3)	86 (53.1)	40 (48.8)	50 (58.1)	36 (63.2)	10 (45.5)	0.391
Hospital duration (days)	43 (23–76)	40 (23–67)	45 (22–76)	35 (22–72)	52 (31–88)	46 (26–71)	0.287
Underlying diseases							
Diabetes mellitus	155 (37.9)	61 (37.7)	28 (34.1)	37 (43.0)	21 (36.8)	8 (36.4)	0.826
Solid tumor	228 (55.7)	86 (53.1)	54 (65.9)	48 (55.8)	28 (49.1)	12 (54.5)	0.299
Hematologic malignancy	20 (4.9)	5 (3.1)	6 (7.3)	3 (3.5)	2 (3.5)	4 (18.2)	0.025
Cerebrovascular diseases	46 (11.2)	14 (8.6)	13 (15.9)	8 (9.3)	11 (19.3)	0	0.047
Chronic lung diseases	28 (6.8)	12 (7.4)	5 (6.1)	5 (5.8)	5 (8.8)	1 (4.5)	0.937
Liver cirrhosis	22 (5.4)	9 (5.6)	3 (3.7)	3 (3.5)	6 (10.5)	1 (4.5)	0.394
Chronic heart failure	21 (5.1)	10 (6.2)	6 (7.3)	3 (3.5)	2 (3.5)	0	0.535
End stage renal diseases	19 (4.6)	6 (3.7)	2 (2.4)	4 (4.7)	6 (10.5)	1 (4.5)	0.225
Solid organ transplantation	2 (0.5)	1 (0.6)	0	1 (1.2)	0	0	0.800
ICU stay at candidemia onset	91 (22.2)	37 (22.8)	8 (9.8)	22 (25.6)	17 (29.8)	7 (31.8)	0.026
Nosocomial candidemia	325 (79.5)	130 (80.2)	58 (70.7)	66 (76.7)	52 (91.2)	19 (86.4)	0.045
Persistent candidemia (n = 334) ^c^	55 (16.5)	16 (13.0)	23 (31.1)	9 (11.8)	6 (14.3)	1 (5.3)	0.004
Candida colonization	102 (24.9)	46 (28.4)	9 (11.0)	22 (25.6)	19 (33.3)	6 (27.3)	0.019
TTP (h) (n = 287) ^c^	24 (14–37)	24 (14–37)	24 (15–34)	36 (24–48)	17 (8–21)	18 (13–27)	<0.001
Concomitant bacteremia	59 (14.4)	22 (13.6)	7 (8.5)	13 (15.1)	14 (24.6)	3 (13.6)	0.126
Charlson comorbidity index	6 (4–6)	6 (4–8)	7 (5–8)	6 (5–8)	7 (4–9)	6 (5–8)	0.877
Surgery	51 (12.5)	20 (12.3)	2 (2.4)	18 (20.9)	9 (15.8)	2 (9.1)	0.007
Gastrointestinal surgery	25 (6.1)	12 (7.4)	1 (1.2)	8 (9.3)	3 (5.3)	1 (4.5)	0.185
Total parenteral nutrition	175 (42.8)	67 (41.4)	39 (47.6)	39 (45.3)	22 (38.6)	8 (36.4)	0.753
Corticosteroid	68 (16.6)	24 (14.8)	16 (19.5)	9 (10.5)	10 (17.5)	9 (40.9)	0.013
Chemotherapy	94 (23.0)	32 (19.8)	29 (35.4)	18 (20.9)	7 (12.3)	8 (36.4)	0.007
Neutropenia (<500/mm^3^)	33 (8.1)	14 (8.6)	8 (9.8)	4 (4.7)	5 (8.8)	2 (9.1)	0.771
Prior antibiotics	343 (83.9)	137 (84.6)	62 (75.6)	71 (82.6)	53 (93.0)	20 (90.9)	0.072
Broad-spectrum antibiotics	273 (66.7)	110 (67.9)	53 (64.6)	54 (62.8)	39 (68.4)	17 (77.3)	0.729
Prior azole exposure	28 (6.8)	6 (3.7)	9 (11.0)	6 (7.0)	3 (5.3)	4 (18.2)	0.053
Mechanical ventilation	56 (13.7)	23 (14.2)	6 (7.3)	13 (15.1)	10 (17.5)	4 (18.2)	0.393
Septic shock	56 (13.7)	29 (17.9)	3 (3.7)	11 (12.8)	10 (17.5)	3 (13.6)	0.037
SOFA score	3 (1–5)	3 (1–6)	2 (0–4)	3.5 (2– 6)	3 (1–7)	3.5 (1–8)	0.020
WBC (×10^3^/mm^3^)	9.1 (5.2–13.6)	10.2 (6.8–14.6)	5.7 (3.5–9.4)	9.4 (5.6–15.2)	10.5 (5.7–13.6)	8.3 (4.9–11.2)	<0.001
CRP (mg/L)	83.7 (40.8–145.0)	92.2 (44.6–151.3)	56.1 (23.5–106.1)	107.6 (55.4–175.5)	85.4 (40.7–153.9)	67.6 (28.5–107.0)	<0.001
CVC in situ	321 (78.5)	129 (79.6)	70 (85.4)	62 (72.1)	41 (71.9)	19 (86.4)	0.149
Primary	242 (59.2)	100 (61.7)	31 (37.8)	55 (64.0)	39 (68.4)	17 (77.3)	<0.001
CRBSI	119 (29.1)	45 (27.8)	49 (59.8)	11 (12.8)	11 (19.3)	3 (13.6)	<0.001
Intra-abdominal	22 (5.4)	4 (2.5)	1 (1.2)	12 (14.0)	4 (7.0)	1 (4.5)	0.001
Urinary tract	24 (5.9)	12 (7.4)	1 (1.2)	8 (9.3)	3 (5.3)	0	0.128
Other	2 (0.5)	1 (0.6)	0	0	0	1 (4.5)	0.072
CVC remove	216 (67.3)	85 (65.9)	60 (85.7)	39 (62.9)	23 (56.1)	9 (47.4)	0.002
CVC remove within 48 h	79 (24.6)	37 (28.7)	24 (34.3)	5 (8.1)	12 (29.3)	1 (5.3)	0.001
Antifungal treatment	351 (85.8)	135 (83.3)	76 (92.7)	75 (87.2)	46 (80.7)	19 (86.4)	0.252
Initial echinocandin	257 (62.8)	96 (59.3)	55 (67.1)	61 (70.9)	30 (52.6)	15 (68.2)	0.150
Initial azole	94 (23.0)	40 (24.7)	19 (23.2)	16 (18.6)	15 (26.3)	4 (18.2)	0.764
Initial amphotericin B	6 (1.5)	2 (1.2)	2 (2.4)	0	2 (3.5%)	0	0.427
Adequate empirical treatment	203 (49.6)	79 (48.8)	52 (63.4)	27 (31.4)	33 (57.9)	11 (50.0)	0.001
COMPLICATON							
Endophthalmitis (n = 154) ^c^	8 (5.1)	6 (3.7)	0	0	2 (3.5)	0	0.131
Bone and joint infections	7 (1.7)	3 (1.9)	1 (1.2)	1 (1.2)	1 (1.8)	1 (4.5)	0.854
Thrombophlebitis	2 (0.5)	0	1 (1.2)	1 (1.2)	0	0	0.577
Hospital mortality	211 (51.6)	88 (54.3)	32 (39.0)	47 (54.7)	29 (50.9)	15 (68.2)	0.077
7-day mortality	88 (21.5)	42 (25.9)	8 (9.8)	15 (17.4)	16 (28.1)	7 (31.8)	0.016
30-day mortality	166 (40.6)	74 (45.7)	21 (25.6)	35 (40.7)	26 (45.6)	10 (45.5)	0.037

Data are presented as no. (%) of patients or median (interquartile range), unless otherwise indicated. Abbreviations: ICU, intensive care unit; TTP, time to blood culture positivity; SOFA, sequential organ failure assessment; WBC, white blood cell; CRP, C-reactive protein; CVC, central venous catheter; CRBSI, catheter-related bloodstream infection. ^a^ Includes *C. guilliermondii* (n = 6), *C. krusei* (n = 5), *C. famata* (n = 3), *C. pelliculosa* (n = 3), *C. lusitaniae* (n = 2), *C. dubliniensis* (n = 2), and *C. utilis* (n = 1). ^b^ The *p*-value represents the comparison across all groups. Refer to Supplementary Table S1 for comparisons between *C. albicans* and the other three *Candida* species, and Supplementary Table S2 for pairwise multiple comparison results. ^c^ Number of patients for whom test results were available.

**Table 3:** Univariate and multivariate Cox regression analyses of predictors for 30-day mortality in patients with *Candida* BSIs.

Variables	Reference Group	HR (95% CI)	*p*	Adjusted HR (95% CI)	*p*
Age		1.00 (0.99–1.02)	0.528		
Female	male	0.80 (0.59–1.09)	0.150		
Underlying diseases					
Diabetes mellitus		0.87 (0.63–1.19)	0.388		
Solid tumor		1.61 (1.17–2.21)	0.004	1.27 (0.64–2.52)	0.486
Hematologic disease		0.56 (0.23–1.35)	0.195		
Cerebrovascular disease		0.61 (0.35–1.08)	0.090	0.72 (0.32–1.59)	0.415
Chronic obstructive pulmonary disease		1.34 (0.76–2.37)	0.308		
Liver cirrhosis		1.98 (1.14–3.42)	0.015	1.02 (0.44–2.37)	0.968
End-stage renal disease		0.72 (0.32–1.63)	0.436		
Chronic heart failure		1.50 (0.82–2.77)	0.191		
Charlson comorbidity index		1.22 (1.14–1.29)	<0.001	1.20 (1.07–1.35)	0.001
ICU stay at onset of candidemia		1.97 (1.41–2.74)	<0.001	1.33 (0.59–3.02)	0.493
Risk factors for *Candida* infection					
*Candida* colonization		1.05 (0.74–1.49)	0.791		
Total parenteral nutrition		1.27 (0.93–1.72)	0.127		
Neutropenia (<500/mm^3^)		1.55 (0.95–2.52)	0.081	1.52 (0.77–3.00)	0.226
Chemotherapy		0.95 (0.66–1.37)	0.800		
Corticosteroid		1.67 (1.16–2.42)	0.006	1.48 (0.71–3.10)	0.301
Surgery		0.63 (0.37–1.08)	0.091	0.56 (0.23–1.36)	0.200
Gastrointestinal surgery		0.74 (0.36–1.51)	0.410		
Central venous catheter in situ		1.26 (0.85–1.88)	0.247		
Mechanical ventilation		2.16 (1.47–3.16)	<0.001	0.72 (0.29–1.76)	0.465
Prior broad-spectrum antibiotics		1.46 (1.04–2.05)	0.029	1.29 (0.80–2.08)	0.292
Persistent candidemia		0.86 (0.50–1.50)	0.599		
SOFA score		1.20 (1.16–1.24)	<0.001	1.12 (1.02–1.23)	0.022
Septic shock		4.91 (3.47–6.94)	<0.001	2.20 (0.99–4.90)	0.052
Source of candidemia					
CRBSI		0.43 (0.29–0.64)	<0.001	1.07 (0.34–3.40)	0.908
Primary		2.25 (1.60–3.16)	<0.001	0.80 (0.28–2.25)	0.671
Gastrointestinal		1.20 (0.63–2.27)	0.585		
Urinary		0.58 (0.26–1.31)	0.189		
*Candida species*	*C. albicans*				
*Candida parapsilosiss*		0.49 (0.30–0.80)	0.004	0.46 (0.22–0.99)	0.047
*Candida glabrata*		0.81 (0.54–1.22)	0.317	0.82 (0.47–1.46)	0.506
*Candida tropicalis*		1.07 (0.69–1.68)	0.754	0.92 (0.48–1.77)	0.800
Others		1.03 (0.53–1.99)	0.934	0.50 (0.19–1.29)	0.151
TTP		0.99 (0.98–1.00)	0.275		
Early CVC removal (≤48 h)		0.38 (0.22–0.66)	0.001	1.26 (0.66–2.40)	0.480
CVC removal at any time		0.16 (0.10–0.24)	<0.001	0.22 (0.13–0.37)	<0.001
First-line echinocandin treatment		1.57 (0.95–2.41)	0.079	1.53 (0.86–2.73)	0.149
Early initial adequate antifungal agent (≤48 h)		0.54 (0.36–0.81)	0.003	0.83 (0.50–1.38)	0.479

Abbreviations: BSI, bloodstream infection; HR, hazard ratio; CI, confidence interval; ICU, intensive care unit; SOFA, sequential organ failure assessment; CRBSI, catheter-related bloodstream infection; TTP, time to blood culture positivity; CVC, central venous catheter.

## Data Availability

The original contributions presented in this study are included in the article and Supplementary Materials. Further inquiries can be directed to the corresponding author.

## Supplementary Materials

The following supporting information can be downloaded at: https://www.mdpi.com/article/10.3390/jof11030217/s1, Figure S1: Ratio of fluconazole non-susceptible Candida isolates from bloodstream infections for which susceptibility tests were available during the study period; Table S1: Comparison of clinical characteristics among patients with bloodstream infections caused by major Candida species; Table S2: Comparison of clinical characteristics among patients with Candida bloodstream infections, including multiple pairwise comparisons.
