# Supplementary material for: Epidemiology and Clinical Features of Candida Bloodstream Infections: A 10-Year Retrospective Study in a Korean Teaching Hospital
Source: J Fungi (Basel). 2025 Mar 12;11(3):217. doi: 10.3390/jof11030217 (PMC11942763; doi:10.3390/jof11030217)
Supplement: Supplementary file 1 [file jof-11-00217-s001.zip › jof-3471943-supplementary.pdf]

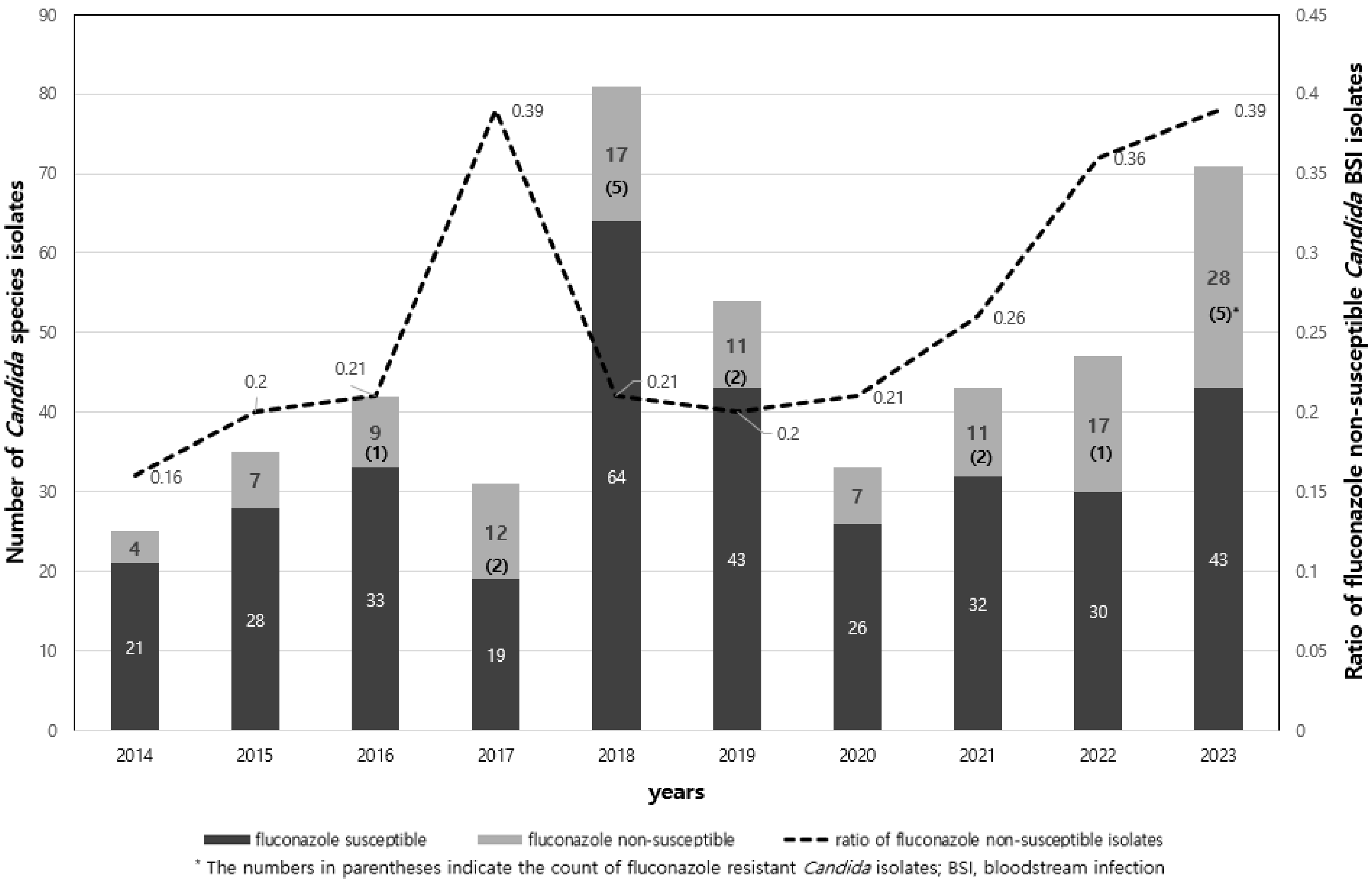

Supplementary Figure S1. Ratio of fluconazole non-susceptible *Candida* isolates from bloodstream infections for which susceptibility tests were available during the study period.

Supplementary Table S1. Comparison of clinical characteristics among patients with bloodstream infections caused by major *Candida* species

Variable	*C. albicans* (CA) (*n* = 162)	*C. parapsilosis* (CP) (*n* = 82)	*C. glabrata* (CG) (*n* = 86)	*C. tropicalis* (CT) (*n* = 57)	*p*		
					CA *vs.* CP	CA *vs.* CG	CA *vs.* CT
Age (years), median (IQR)	70 (62–78)	71 (61–79)	74 (63–80)	68 (58–75)	0.617	0.271	0.265
Male	86 (53.1)	40 (48.8)	50 (58.1)	36 (63.2)	0.617	0.531	0.245
Hospital duration (days), median(IQR)	40 (23–67)	45 (22–76)	35 (22–72)	52 (31–88)	0.710	0.525	0.066
Underlying diseases							
Diabetes mellitus	61 (37.7)	28 (34.1)	37 (43.0)	21 (36.8)	0.691	0.492	0.999
Solid tumor	86 (53.1)	54 (65.9)	48 (55.8)	28 (49.1)	0.077	0.782	0.718
Hematologic malignancy	5 (3.1)	6 (7.3)	3 (3.5)	2 (3.5)	0.239	0.999	0.999
Cerebrovascular diseases	14 (8.6)	13 (15.9)	8 (9.3)	11 (19.3)	0.139	0.999	0.053
Chronic lung diseases	12 (7.4)	5 (6.1)	5 (5.8)	5 (8.8)	0.910	0.835	0.965
Liver cirrhosis	9 (5.6)	3 (3.7)	3 (3.5)	6 (10.5)	0.738	0.681	0.331
Chronic heart failure	10 (6.2)	6 (7.3)	3 (3.5)	2 (3.5)	0.946	0.546	0.673
End stage renal diseases	6 (3.7)	2 (2.4)	4 (4.7)	6 (10.5)	0.886	0.983	0.108
Solid organ transplantation	1 (0.6)	0	1 (1.2)	0	0.999	0.999	0.999
ICU stay at candidemia onset	37 (22.8)	8 (9.8)	22 (25.6)	17 (29.8)	0.021	0.744	0.382
Nosocomial candidemia	130 (80.2)	58 (70.7)	66 (76.7)	52 (91.2)	0.131	0.630	0.090
Persistent candidemia (*n* = 334) [a]	16 (13.0)	23 (31.1)	9 (11.8)	6 (14.3)	0.004	0.983	0.999
Candida colonization	46 (28.4)	9 (11.0)	22 (25.6)	19 (33.3)	0.004	0.747	0.594

TTP (h) (*n* = 287) [a]	24 (14–37)	24 (15–34)	36 (24–48)	17 (8–21)	0.836	0.001	0.001
Concomitant bacteremia	22 (13.6)	7 (8.5)	13 (15.1)	14 (24.6)	0.347	0.889	0.086
Charlson comorbidity index	6 (4–8)	7 (5–8)	6 (5–8)	7 (4–9)	0.335	0.937	0.825
Surgery	20 (12.3)	2 (2.4)	18 (20.9)	9 (15.8)	0.021	0.109	0.665
Gastrointestinal surgery	12 (7.4)	1 (1.2)	8 (9.3)	3 (5.3)	0.083	0.782	0.805
Total parenteral nutrition	67 (41.4)	39 (47.6)	39 (45.3)	22 (38.6)	0.432	0.639	0.835
Corticosteroid	24 (14.8)	16 (19.5)	9 (10.5)	10 (17.5)	0.451	0.445	0.782
Chemotherapy	32 (19.8)	29 (35.4)	18 (20.9)	7 (12.3)	0.012	0.957	0.286
Neutropenia (< 500/mm^3)	14 (8.6)	8 (9.8)	4 (4.7)	5 (8.8)	0.960	0.370	0.999
Prior antibiotics	137 (84.6)	62 (75.6)	71 (82.6)	53 (93.0)	0.126	0.820	0.166
Broad antibiotics	110 (67.9)	53 (64.6)	54 (62.8)	39 (68.4)	0.713	0.504	0.999
Prior azole exposure	6 (3.7)	9 (11.0)	6 (7.0)	3 (5.3)	0.051	0.405	0.903
Mechanical ventilation	23 (14.2)	6 (7.3)	13 (15.1)	10 (17.5)	0.174	0.995	0.695
Septic shock	29 (17.9)	3 (3.7)	11 (12.8)	10 (17.5)	0.004	0.390	0.999
SOFA score	3 (1–6)	2 (0–4)	3.5 (2– 6)	3 (1–7)	0.012	0.613	0.348
WBC (×10^3/mm^3)	10.2 (6.8–14.6)	5.7 (3.5–9.4)	9.4 (5.6–15.2)	10.5 (5.7–13.6)	<0.001	0.667	0.579
CRP (mg/L)	92.2 (44.6–151.3)	56.1 (23.5–106.1)	107.6 (55.4–175.5)	85.4 (40.7–153.9)	<0.001	0.243	0.654
CVC *in situ*	129 (79.6)	70 (85.4)	62 (72.1)	41 (71.9)	0.359	0.236	0.310
Primary	45 (27.8)	49 (59.8)	11 (12.8)	11 (19.3)	0.347	<0.001	0.015
CRBSI	45 (27.8)	49 (59.8)	11 (12.8)	11 (19.3)	<0.001	0.012	0.278
Intra-abdominal	4 (2.5)	1 (1.2)	12 (14.0)	4 (7.0)	0.863	0.001	0.244
Urinary tract	12 (7.4)	1 (1.2)	8 (9.3)	3 (5.3)	0.063	0.782	0.805

Other	1 (0.6)	0	0	0	0.999	0.999	0.999
CVC remove	85 (65.9)	60 (85.7)	39 (62.9)	23 (56.1)	0.005	0.808	0.343
CVC remove within 48h	37 (28.7)	24 (34.3)	5 (8.1)	12 (29.3)	0.511	0.002	0.999
Antifungal treatment	135 (83.3)	76 (92.7)	75 (87.2)	46 (80.7)	0.069	0.534	0.804
Initial echinocandin	96 (59.3)	55 (67.1)	61 (70.9)	30 (52.6)	0.295	0.094	0.475
Initial azole	40 (24.7)	19 (23.2)	16 (18.6)	15 (26.3)	0.917	0.352	0.267
Initial amphotericin	2 (1.2)	2 (2.4)	0 (0.0%)	2 (3.5%)	0.868	0.773	0.142
Adequate empirical treatment	79 (48.8)	52 (63.4)	27 (31.4)	33 (57.9)	0.052	0.010	0.341
COMPLICATON							
Endophthalmitis (*n* = 154) [a]	6 (3.7)	0	0	2 (3.5)	0.185	0.170	0.999
Bone and joint infections	3 (1.9)	1 (1.2)	1 (1.2)	1 (1.8)	0.999	0.999	0.999
Thrombophlebitis	0	1 (1.2)	1 (1.2)	0	0.728	0.747	–
Hospital mortality	88 (54.3)	32 (39.0)	47 (54.7)	29 (50.9)	0.034	0.999	0.769
7-day mortality	42 (25.9)	8 (9.8)	15 (17.4)	16 (28.1)	0.005	0.176	0.888
30-day mortality	74 (45.7)	21 (25.6)	35 (40.7)	26 (45.6)	0.004	0.537	0.999

Data are presented as no. (%) of patients or median (interquartile range), unless otherwise indicated. Abbreviations: ICU, Intensive care unit; TTP, time to blood culture positivity; SOFA, sequential organ failure assessment; WBC, white blood cell; CRP, C-reactive protein; CVC, central venous catheter; CRBSI, catheter-related bloodstream infection. [a] Number of patients for whom test results were available.

Supplementary Table S2. Comparison of clinical characteristics among patients with *Candida* bloodstream infections, including multiple pairwise comparisons.[a]

Variable	Total (n = 409)	*C. albicans* (CA) (n = 162)	*C. parapsilosis* (CP) (n = 82)	*C. glabrata* (CG) (n = 86)	*C. tropicalis* (CT) (n = 57)	Others[a] (CO) (n = 22)	*p* [b]	**Pairwise comparisons *p* [a]**
ICU stay at candidemia onset	91 (22.2)	37 (22.8)	8 (9.8)	22 (25.6)	17 (29.8)	7 (31.8)	0.026	**CP *vs.* CT: 0.005**
Persistent candidemia (*n* = 334) [c]	55 (16.5)	16 (13.0)	23 (31.1)	9 (11.8)	6 (14.3)	1 (5.3)	0.004	**CA *vs.* CP: 0.004**
Candida colonization	102 (24.9)	46 (28.4)	9 (11.0)	22 (25.6)	19 (33.3)	6 (27.3)	0.019	**CA *vs.*CP: 0.004**<br>**CP *vs.* CT: 0.003**
TTP (h) (*n* = 287) [c]	24 (14–37)	24 (14–37)	24 (15–34)	36 (24–48)	17 (8–21)	18 (13–27)	<0.001	**CA *vs.* CG: 0.001**<br>**CA *vs.* CT: <0.001**<br>**CP *vs.* CG: <0.001**
Surgery	51 (12.5)	20 (12.3)	2 (2.4)	18 (20.9)	9 (15.8)	2 (9.1)	0.007	**CP *vs.* CG: 0.001**
Corticosteroid	68 (16.6)	24 (14.8)	16 (19.5)	9 (10.5)	10 (17.5)	9 (40.9)	0.013	**CG *vs.* CO: 0.002**
Chemotherapy	94 (23.0)	32 (19.8)	29 (35.4)	18 (20.9)	7 (12.3)	8 (36.4)	0.007	**CP *vs.* CT: 0.004**
Septic shock	56 (13.7)	29 (17.9)	3 (3.7)	11 (12.8)	10 (17.5)	3 (13.6)	0.037	**CA *vs.* CP: 0.004**
WBC (×10^3/mm^3)	9.1 (5.2–13.6)	10.2 (6.8–14.6)	5.7 (3.5–9.4)	9.4 (5.6–15.2)	10.5 (5.7–13.6)	8.3 (4.9–11.2)	<0.001	**CA *vs.* CP: <0.001**<br>**CP *vs.*CG: <0.001**<br>**CP *vs.* CT: <0.001**
CRP (mg/L)	83.7 (40.8–145.0)	92.2 (44.6–151.3)	56.1 (23.5–106.1)	107.6 (55.4–175.5)	85.4 (40.7–153.9)	67.6 (28.5–107.0)	<0.001	**CA *vs.* CP: <0.001**<br>**CP *vs.* CG: <0.001**
CRBSI	119 (29.1)	45 (27.8)	49 (59.8)	11 (12.8)	11 (19.3)	3 (13.6)	<0.001	**CA *vs.* CP: <0.001**<br>**CP *vs.* CG: <0.001**<br>**CP *vs.* CT: <0.001**<br>**CP *vs.* CO: <0.001**

Primary	242 (59.2)	100 (61.7)	31 (37.8)	55 (64.0)	39 (68.4)	17 (77.3)	<0.001	**CA *vs*. CP: <0.001**<br>**CP *vs*. CG: 0.001**<br>**CP *vs*. CT: <0.001**<br>**CP *vs*. CO: 0.002**
Intra-abdominal	22 (5.4)	4 (2.5)	1 (1.2)	12 (14.0)	4 (7.0)	1 (4.5)	0.001	**CP *vs*. CG: 0.005**
CVC remove	216 (67.3)	85 (65.9)	60 (85.7)	39 (62.9)	23 (56.1)	9 (47.4)	0.002	**CA *vs*. CP: 0.003**<br>**CP *vs*. CG: <0.001**<br>**CP *vs*. CT: <0.001**
CVC remove within 48h	79 (24.6)	37 (28.7)	24 (34.3)	5 (8.1)	12 (29.3)	1 (5.3)	0.001	**CA *vs*. CG: 0.001**<br>**CP *vs*. CG: <0.001**
Adequate empirical treatment	203 (49.6)	79 (48.8)	52 (63.4)	27 (31.4)	33 (57.9)	11 (50.0)	0.001	**CP *vs*. CG: <0.001**<br>**CG *vs*. CT: 0.003**
7-day mortality	88 (21.5)	42 (25.9)	8 (9.8)	15 (17.4)	16 (28.1)	7 (31.8)	0.016	**CA *vs*. CP: 0.005**
30-day mortality	166 (40.6)	74 (45.7)	21 (25.6)	35 (40.7)	26 (45.6)	10 (45.5)	0.037	**CA *vs*. CP: 0.004**

Data are presented as no. (%) of patients or median (interquartile range), unless otherwise indicated. Abbreviations: ICU, Intensive care unit; TTP, time to blood culture positivity; WBC, white blood cell; CRP, C-reactive protein; CVC, central venous catheter; CRBSI, catheter-related bloodstream infection. [a] Only statistically significant results from multiple pairwise comparisons are shown. ($P < 0.005$ is considered statistically significant based on Bonferroni adjustment.). [b] The *P*-value represents the comparison among all groups and were obtained using the chi-square test or Kruskal-Wallis test, as appropriate for each variable. [c] Number of patients for whom test results were available.
